# 

**Published:** 1919-12-13

**Authors:** 


### THE COLLEGE OF NURSING (Limited by Guarantee).

DUNDEE CENTRE.

Under the organisation of Miss Pegg, Matron of the Royal Infirmary, Dundee, it was decided at the meeting held on March 27 last that a centre of the College of Nursing for Dundee and district should be formed. Miss Gill, Matron of the Royal Infirmary, Edinburgh, gave a most interesting address to a large gathering of nurses in May. She gave the history of the College from its beginning, emphasised the important activities it had started for the nursing profession, and dwelt upon the advantages of local centres. Her great point properly was that any association of nurses must be what the members themselves made it, and this was especially true of the College of Nursing, which all fully-trained nurses should join without delay.

Mrs. J. C. Buist is the President of the Dundee Centre, and she has already shown marked interest and brought important influence to bear upon its development. The Centre has properly determined to have a Finance Committee, which will be charged with the duty of pursuing definite objects and of working them out to the fullest extent possible. Lady Cowdray, with her usual generosity, has offered £100, provided others will contribute collectively £300 more, to enable the establishment of College of Nursing scholarships for Dundee nurses. We are glad to be able to report, as showing the importance of the Finance Committee, and the activity of those responsible for the Centres of the College of Nursing, that £500 has been granted by the Red Cross Society to the Dundee Centre, and the money is safely in the hands of Mrs. Kydd, their treasurer. We offer Mrs. Kydd our warm congratulations on this splendid result of her efforts, learning that this generous gift has been given largely in her honour for her work during the war. Arrangements are being made to call a large public meeting in Dundee to stimulate interest in the College, when it is hoped that Sir Arthur Stanley will deliver an address. Meanwhile good progress is being made. In March last only sixty members of the College resided in Dundee and district. At the end of November the number of members of the College had increased to 148. Miss Gill's eloquence caused 39 members to join the Dundee Centre, which had a total of 49 members before the annual meeting held last Friday, the 5th inst., when there were 60 members present.

The names of the Committee are: Miss Pegg, Royal Infirmary (Chairman); Mrs. Kydd, late Caird Red Cross Hospital (Treasurer); Miss Clark, King's Cross Hospital (Secretary); Miss Barnett, Royal Infirmary; Miss Boath, late War Hospital; Miss Chapman, Central Nursing Home; Miss E. A. Clark, Women's Hospital; Miss Little, Victoria Hospital; Miss Martin, Infant Hospital; Miss Milne, Queen's Nurses' Association.

### Matrons' and Sisters' Appointments.

COTTAGE HOSPITAL, HENDON.—Miss G. Garland has been appointed theatre and ward sister. She was trained at the London Hospital, later being staff nurse there, and she has done military nursing as sister, home sister, and night superintendent.

FEVER HOSPITAL, WEST BROMWICH.—Miss Mary Fox has been appointed sister. She was trained at Birmingham General Hospital, and has since done private nursing, and has been charge sister and night superintendent in Queen Alexandra's Imperial Military Nursing Service Reserve.

KENSINGTON INFIRMARY.—Miss Elizabeth Muirden and Mrs. Jervis Holloway have been appointed sisters. They were both trained at the above-named institution, and Miss Muirden has since been staff nurse. Miss Muirden and Mrs. Holloway both hold the certificate of the Central Midwives Board.

MUNICIPAL TUBERCULOSIS HOSPITAL, NORTHAMPTON.—Miss A. Allen Patrick has been appointed sister. She was trained at Sulcoates Infirmary, Hull, has served with the Territorial Force Nursing Service as sister at the 1st Northern General Hospital, Newcastle-on-Tyne, in France, and with the Army of Occupation on the Rhine.

ST. MARY ISLINGTON INFIRMARY, HIGHGATE HILL.—Miss Alice Carter and Miss Alice Walpole have been appointed night superintendents, Miss Alice Aylward, R.R.C., sister, and Miss Rebecca Draper, R.R.C., relief home sister respectively. Miss Carter was trained at Chelsea Infirmary, was later ward sister there and at the St. Mary Islington Infirmary, and has been staff nurse in Queen Alexandra's Imperial Military Nursing Service. Miss Walpole was trained at St. Mary Islington Infirmary, later being ward sister there, has been a Queen's nurse, and has served as sister in Queen Alexandra's Imperial Military Nursing Service Reserve. Miss Aylward was trained at Whitechapel Infirmary, has been staff nurse at St. Mary Islington Infirmary, and has served as sister in Queen Alexandra's Imperial Military Nursing Service Reserve. Miss Draper was trained at Mill Road Infirmary, Liverpool, later being ward and theatre sister there; has been sister at Nottingham Eye Hospital, and has served as sister in the Territorial Force Nursing Service both at home and abroad.

ROYAL HOSPITAL FOR SICK CHILDREN, EDINBURGH.—Miss Florence Pemberton has been appointed ward sister. She was trained at the Royal Waterloo Hospital for Women and Children and the Seamen's Hospital, Greenwich, has served as sister in Queen Alexandra's Imperial Military Nursing Service Reserve, and has been on the private staff of the Royal Sussex County Hospital.

STOBHILL HOSPITAL, GLASGOW.—Miss Florence Ada Merchant has been appointed matron. She was trained at the Warneford Hospital, Leamington Spa, and holds the certificates of the Scottish Board of Health, of the Medico-Psychological Association, of the Central Midwives Board, and the Incorporated Sanitary Association of Scotland. She has been matron of the Eastern District Hospital, Glasgow.

